# The complete mitochondrial genome of a wild edible mushroom, *Russula griseocarnosa*

**DOI:** 10.1080/23802359.2019.1674215

**Published:** 2019-10-04

**Authors:** Fei Yu, Yongjie Zhang, Jie Song, Junfeng Liang

**Affiliations:** a Key Laboratory of State Forestry Administration on Tropical Forestry Research, Research Institute of Tropical Forestry, Chinese Academy of Forestry, Guangzhou, China;; b College of Life Science, Shanxi University, Taiyuan, China

**Keywords:** *Russula griseocarnosa*, mitochondrion, phylogenetic analysis

## Abstract

*Russula griseocarnosa* is a wild edible ectomycorrhizal mushroom in southern China. In this study, we assembled the complete mitochondrial genome of *R. griseocarnosa*. Its total length was 60995 bp with a GC content of 21% and contained a total of 52 genes, including 14 standard protein-coding genes, two rRNA genes, 21 tRNA genes and 15 free-standing open reading frames (ORFs). Phylogenetic analysis reflected that the evolutionary processes between *R. griseocarnosa* and some agaricomycetes.

*Russula griseocarnosa* is an important wild edible mycorrhizal fungus in southern China (Wang et al. 2009). Previous studies confirmed that the chemical properties played a great role in the antioxidant and anti-tumor activities of *R. griseocarnosa* (Chen et al. 2010; Chen et al. 2018; Liu et al. 2018; Yu et al. 2019). Mitochondria are presumed to be derived from bacteria through endosymbiosis (Muñoz-Gómez et al). Many mitochondrial genomes (mitogenomes) contribute to systemic evolution, population genetics, and taxonomy (Carpi et al. 2016; Ramos et al. 2018). MtDNA analysis has been carried out for six species of *Russula* (Li et al. 2018), but the mitochondrial genome of *R. griseocarnosa* has not been reported.

Fruiting bodies of *R. griseocarnosa* (strain LJ24) were collected from Linjing Town, Teng County, Guangxi Province, China (110°38′E, 23°8′N), frozen rapidly in liquid nitrogen, brought back to the laboratory and placed at −80 °C in the Research Institute of Tropical Forestry, Chinese Academy of Forestry. The genomic DNA was extracted with the Omega Fungal DNA Kit D3390-02, and then sequenced on Illumina HiSeq X-ten sequencing platform at Shanghai Majorbio Bio-pharm Biotechnology Co., Ltd, China.

The assembly of the mitogenome of *R.griseocarnosa* were carried out using MITObim V1.9.1 (Hahn et al. 2013) by using the mitogenome of *Russula compacta* (MH138072) as the initial reference. The complete mitogenome was annotated with the MFannot V1.33 (Valach et al. 2014) and MITOS2 (Bernt et al. 2013). Protein coding gene and ORFs were corrected using the NCBI open reading frame finder (https://www.ncbi.nlm.nih.gov/orffinder). tRNA genes were predicted using tRNAscan-SE 2.0 (Lowe and Chan 2016). Individual sequences of 14 protein-coding genes (*atp6*, *atp8*, *atp9*, *cob*, *cox1*,*cox2*, *cox3*, *nad1*, *nad2*, *nad3*, *nad4*, *nad4L*, *nad5* and *nad6*) were aligned using MEGA v6.06, concatenated using SequenceMatrix v1.8, and then a neighbor-joining phylogenetic tree was constructed using MEGA v6.06 with 1000 bootstrap replicates.

The mitogenome of *Russula griseocarnosa* was assembled as a 60995-bp circular molecule with a GC content of 21% (GenBank accession No. MN427435). It contains 52 genes, including 14 protein-coding genes, two rRNA genes, 21 tRNA genes and 15 open reading frames (ORFs). The start codon of 14 protein coding gene was ATG, and the termination codon was TAA. The *R. griseocarnosa* mitogenome contained two tRNA genes with different anticodons for leucine and serine, and three tRNAs with the same anticodons for methionine. The mitogenome of *R. griseocarnosa* contains six introns, which are located at *nad5* (1 intron), *cob* (2 introns) and *cox1* (3 introns).

To examine the phylogenetic evolution of *R. griseocarnosa*, a neighbor-joining phylogenetic analysis was applied for 14 agaricomycetes mitogenomes (including *R. griseocarnosa*) based on 14 protein-coding genes and rooted with *Neurospora crassa* (Figure 1). The phylogenetic evolution indicats a close relationship between *R. griseocarnosa* and other *Russula*. It will help us to understanding the evolutionary processes.

**Figure 1.**
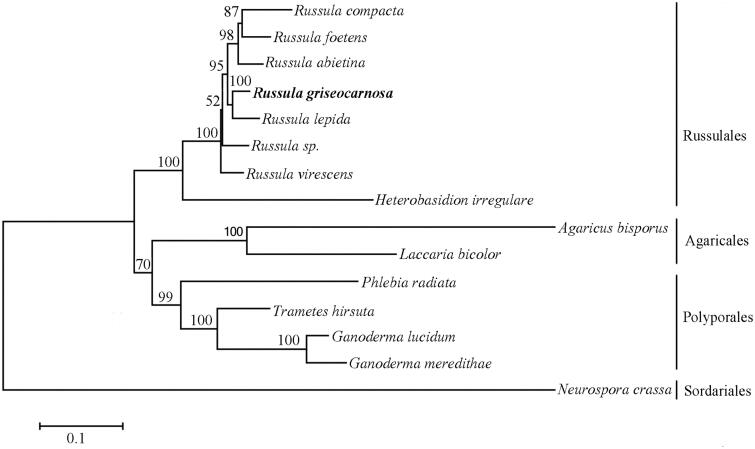
Phylogenetic analyses of 15 fungi based on 14 protein coding genes. GenBank accessionn numbers: *Agaricus bisporus* (JX271275.1), *Ganoderma lucidum* (NC_021750.1), *Ganoderma meredithae* (NC_026782.1), *Heterobasidion irregulare* (KF957635.1), *Laccaria bicolour* (NC_042773.1), *Phlebia radiata* (HE613568.1), *Russula abietina* (MH138073.1), *Russula compacta* (MH138072.1), *Russula foetens* (MH138074.1), *Russula lepida* (MH138075.1), *Russula sp.* (MH138077.1), *Russula virescens* (MH138076.1), *Trametes hirsuta* (MG775432.1) and *Neurospora crassa* (KY498477.1).
